# Supplementary material for: Measures of Adrenal and Gonadal Hormones in Relation to Biological and Management Factors among Captive Red Pandas in Indian Zoos
Source: Animals (Basel). 2023 Apr 10;13(8):1298. doi: 10.3390/ani13081298 (PMC10135066; doi:10.3390/ani13081298)
Supplement: Supplementary file 1 [file animals-13-01298-s001.zip › animals-2145399-supplementary.pdf]

animals

MDPI

Article

Measures of Adrenal and Gonadal Hormones in Relation to Biological and Management Factors among Captive Red Pandas in Indian Zoos

Aamer Sohel Khan [1], Janine Brown [2], Vinod Kumar [3], Govindhaswamy Umapathy [3] and Nagarajan Baskaran [1,*]

[1] Mammalian Biology Lab, Department of Zoology and Wildlife Biology, Anbanathpuram Vahaira Charity (A.V.C.) College (Autonomous), Mannampandal, Mayiladuthurai 609305, Tamil Nadu, India; khannaamirsohel@gmail.com
[2] Smithsonian National Zoo and Conservation Biology Institute, Center for Species Survival, Front Royal, VA 22630, USA; brownjan@si.edu
[3] Laboratory for the Conservation of Endangered Species, CSIR-Center for Cellular and Molecular Biology, Habsiguda, Hyderabad 500007, Telangana, India; vinod@ccmb.res.in (V.K.); guma@ccmb.res.in (G.U.)
* Correspondence: nagarajan.baskaran@gmail.com; Tel.: +91-4364-8903410794

Supplementary Table S1: Red pandas sampled in three zoos, their demographic and enclosures details.

Place	Red Panda ID	Age	Age class/sex	Sociality	Breeding history	Enclosure area (m^2)	Enclosure type*	Nest No.	Individuals sampled for fGCM/fAM or fPM
Zoo 1	RP1	4	AF	Paired	No	3068	Open	2	Yes / Yes
Zoo 1	RP2	6	AF	Paired	No	192	Open	3	Yes / Yes
Zoo 1	RP3	17	AM	Paired	Yes	192	Open	3	Yes / Yes
Zoo 1	RP4	3	AM	Paired	Yes	1560	Open	2	Yes / Yes
Zoo 1	RP5	5	AF	Paired	Yes	1560	Open	2	Yes / Yes
Zoo 1	RP6	5	AM	Paired	Yes	1560	Open	2	Yes / Yes
Zoo 1	RP7	8	AF	Paired	Yes	495	Open	3	Yes / Yes
Zoo 1	RP8	3	AF	Paired	Yes	2925	Open	3	Yes / Yes
Zoo 1	RP9	7	AF	Female with one cub	Yes	2925	Open	3	Yes / Yes
Zoo 1	RP10	0.6	CF	Female with one cub	-	2925	Open	3	Yes / No
Zoo 1	RP11	3	AM	Solitary	No	350	Open	1	Yes / Yes
Zoo 1	RP12	11	AF	Female with two cubs	Yes	322	Open	3	Yes / Yes
Zoo 1	RP13	0.8	CF	Female with two cubs	-	322	Open	3	Yes / No
Zoo 1	RP14	0.8	CM	Female with two cubs	-	322	Open	3	Yes / No
Zoo 1	RP15	8	AF	Paired	No	462	Open	2	Yes / Yes
Zoo 1	RP16	8	AM	Paired	Yes	462	Open	2	Yes / Yes
Zoo 1	RP17	3	AM	Paired	Yes	400	Open	3	Yes / Yes
Zoo 2	RP18	7	AF	Female with two cubs	Yes	2463	Open	1	Yes / Yes
Zoo 3	RP19	16	AM	Paired	Yes	224	Closed	4	Yes / Yes
Zoo 3	RP20	4	AF	Paired	Yes	224	Closed	4	Yes / Yes

*Open – enclosures were only fenced with open top, closed – enclosures were netted from all sides.

Supplementary Table S2: Details of female's breeding rate since age of maturity and their fGCM and fPM concentration during the study

ID (Age)	Mean fGCM ng/g	Mean fPM µg/g	Breeding rate (a/b*100)[#]
RP1 (4)	47	0.573	0
RP2 (6)	62	0.069	0
RP5 (5)	32	0.262	25
RP7 (8)	41	0.310	42.9
RP8 (3)	17	0.362	100
RP9 (7)	27	0.554	33.3
RP12 (11)	27	0.756	33.3
RP15 (8)	53	1.303	0
RP20 (4)	38	17.986	75

[#]a – Number of liters produced since age of maturity, b – Total breeding age
